# Inhibiting the TGF-β1 Pathway Reduces the Aggressiveness of Intrahepatic CCA HuCCT1 CD90-Positive Cells

**DOI:** 10.3390/ijms26114973

**Published:** 2025-05-22

**Authors:** Elena Pizzuto, Serena Mancarella, Isabella Gigante, Grazia Serino, Francesco Dituri, Emanuele Piccinno, Isabel Fabregat, Gianluigi Giannelli

**Affiliations:** 1 Personalized Medicine Laboratory, National Institute of Gastroenterology “S. de Bellis” IRCCS Research Hospital, 70013 Castellana Grotte, Italy; elena.pizzuto@irccsdebellis.it (E.P.); serena.mancarella@irccsdebellis.it (S.M.); isabella.gigante@irccsdebellis.it (I.G.); grazia.serino@irccsdebellis.it (G.S.); francesco.dituri@irccsdebellis.it (F.D.); emanuele.piccinno@irccsdebellis.it (E.P.); 2 TGF-β and Cancer Group—Oncobell Program, Bellvitge Biomedical Research Institute (IDIBELL), 08860 L’Hospitalet de Llobregat, Spain; ifabregat@idibell.cat; 3 National Biomedical Research Institute on Liver and Gastrointestinal Diseases (CIBEREHD), Instituto de Salud Carlos III, 28029 Madrid, Spain

**Keywords:** intrahepatic cholangiocarcinoma, CD90, TGF-β1 signaling pathway, transcriptomic analysis

## Abstract

Molecular mechanisms responsible for the poor prognosis in patients with intrahepatic cholangiocarcinoma (CCA) are still unknown, but stem cell marker Cluster Differentiation 90 (CD90) has been reported to be associated with a more aggressive cancer phenotype. In this scenario, the TGF-β1 signaling pathway likely has a role as master gene regulator. Aim of the study is to investigate the role of CD90 in iCCA aggressiveness. The molecular profile of HuCCT1/CD90+ and HuCCT1/CD90− cells was obtained through transcriptomic analysis (NGS). Bioinformatic data were confirmed in both cell lines by qRT-PCR and Western blot. Cells were treated with Gemcitabine in monotherapy or in combination with Galunisertib, a selective inhibitor of TGF-βRI, in 2D and 3D models. HuCCT1/CD90+ cells are more proliferative, less migratory, and resistant to Gemcitabine treatment. HuCCT1/CD90+ cells also express lower levels of TGF-β1 compared to /CD90− cell lines. Finally, HuCCT1/CD90+ cells are resistant to Gemcitabine, while the combination of Gemcitabine and Galunisertib displays a synergistic effect on HuCCT1/CD90+ cell proliferation. These results underline that CD90-induced Gemcitabine resistance can be overcome by adding a TGFβ1 inhibitor such as Galunisertib, thereby moving further toward a precision medicine approach in patients with iCCA.

## 1. Introduction

Intrahepatic cholangiocarcinoma (iCCA) accounts for 10–20% of all liver cancers [1]. In the last forty years, the incidence of iCCA has increased in many countries, depending on different risk factors typical of each region [2,3]. Treatment options for iCCA patients are still limited [4]. Nowadays, surgical resection is the only curative treatment available for patients with early-stage disease. Unfortunately, most patients with iCCA are diagnosed at an advanced or metastatic stage. In such cases, the standard treatment involves the combination of two well-known chemotherapeutic agents, Gemcitabine and Cisplatin, as first-line treatment [5,6], yielding only partial benefit. Currently, despite significant advances in diagnostic and therapeutic approaches, the prognosis of iCCA remains poor [7]. This is mainly due to the limited knowledge of iCCA carcinogenic mechanisms and the lack of reliable biomarkers for early diagnosis, prognosis, and treatment response [8]. Therefore, molecular biomarkers are urgently needed to improve clinical outcomes and enable personalized therapeutic strategies. In the last decade, independent groups have reported an overexpression of the glycoprotein Cluster Differentiation 90 (CD90) in several cancers, including melanoma, pancreatic adenocarcinoma (PDA), hepatocellular carcinoma (HCC), and ovarian cancer [9,10,11,12]. CD90 is a surface glycoprotein expressed on different cell types, including stem cells, neurons, endothelial cells, and cancer-associated fibroblasts (CAFs), whose properties are involved in cell–cell and cell–matrix interactions, cytoskeletal organization, migration, and inflammation [13]. Moreover, HCC cells positive for CD90 exhibit tumorigenic and metastatic features, contributing to a poor prognosis [14]. Besides CD90, another historical driver of tumor progression, particularly in the liver, is Transforming Growth Factor-beta 1 (TGF-β1) [15,16]. The activation of TGF-β1 signaling follows the canonical (SMAD-dependent) or non-canonical (SMAD-independent) pathway [17,18]. In the liver, TGF-β1 plays a pivotal role in tumor progression by promoting the epithelial–mesenchymal transition (EMT) and remodeling the cellular phenotype, leading to enhanced invasion and migration properties [19,20]. Galunisertib (LY2157299), an oral small-molecule inhibitor of TGF-β receptor I, effectively reduces phosphorylation of p-Smad2 and inhibits invasion in in vitro HCC models, without affecting cell proliferation [21]. This selective inhibitor has demonstrated promising efficacy as a second-line treatment for a subset of HCC patients in a phase 2 clinical trial [22,23].

Recently, in our research, we found that overexpression of the NOTCH1/HES1/CD90 axis in iCCA patients is associated with a less favorable prognosis. However, data obtained in the in vivo study suggested that inhibiting the Notch signaling pathway can be particularly effective in cases with aberrant CD90 expression. In this context, CD90 represents a potential therapeutic target for iCCA patients [24].

Nevertheless, no data are currently available to explain the molecular aspects of CD90 in iCCA. This study aims to investigate the molecular profile of iCCA HuCCT1/CD90+ cells and the interaction with TGF-β1.

## 2. Results

HuCCT1/CD90+ have been previously reported to display a more aggressive phenotype in iCCA experimental models as well as in patients, being correlated with a shorter survival and worse prognosis [24]. Herein, we investigated the molecular mechanism underlying such different biological behavior in in vitro experimental models.

### 2.1. CD90 Affects the Morphology and Phenotype of HUCCT1 Cells

**Figure 1:**
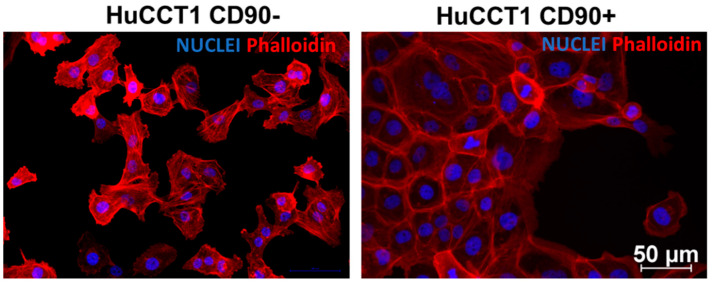
Phalloidin TRITC and DAPI co-staining in HuCCT1 CD90−/CD90+. Rhodamine Phalloidin TRITC shows a high affinity for F-actin and is therefore used to label actin filaments in immunofluorescence studies. Phalloidin staining shows a distinct morphology between the two cell lines, showing strong cell contact in CD90+ cells. (Magnification: 20×; Scale bar 50 µM for both images).

HuCCT1/CD90− and /CD90+ cells grow differently, as shown in Figure 1. The first cell line shows an islet-like structure, while the second has an epithelial-sheet-like structure.

**Figure 2:**
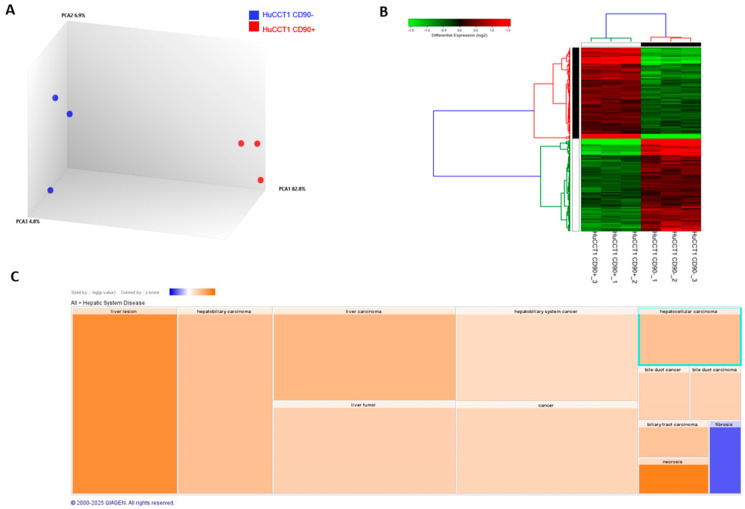
Transcriptomic profiling of HuCCT1 CD90−/CD90+ cell line. Principal Component Analysis (**A**) and hierarchical clustering heatmap (**B**) using differential expressed genes show a clear separation between the two analyzed cell lines. (**C**). IPA using the annotations “Disease & Function” revealed the most genes in Hepatic System Disease. Results are visualized as a hierarchical heatmap, where the boxes represent a category of related functions. Each colored rectangle represents a particular biological function or disease, and the color indicates the state of prediction: increase (orange) and decrease (blue).

To further characterize the biological and molecular differences between HuCCT1/CD90+ and /CD90− cells, we investigated the whole transcriptomic profile by NGS. Applying a ±1.5 cut-off fold-change threshold, we identified 4016 differentially expressed genes (DEGs) (1996 up and 2020 down). A clear separation between the HuCCT1/CD90+ and HuCCT1/CD90− was observed, as revealed by Principal Component Analysis (PCA) (Figure 2A). The difference was further confirmed by unsupervised hierarchical analysis clustering (HC) (Figure 2B). To investigate more deeply the biological effects of DEGs, we conducted an IPA core analysis for “Disease & Function”. This bioinformatic analysis demonstrated that the highest number of genes was present in Hepatic System Diseases, particularly in liver lesions and hepatobiliary cancer (Figure 2C).

### 2.2. Effects of CD90 Overexpression on Proliferation and Migration of HUCCT1 Cells

**Figure 3:**
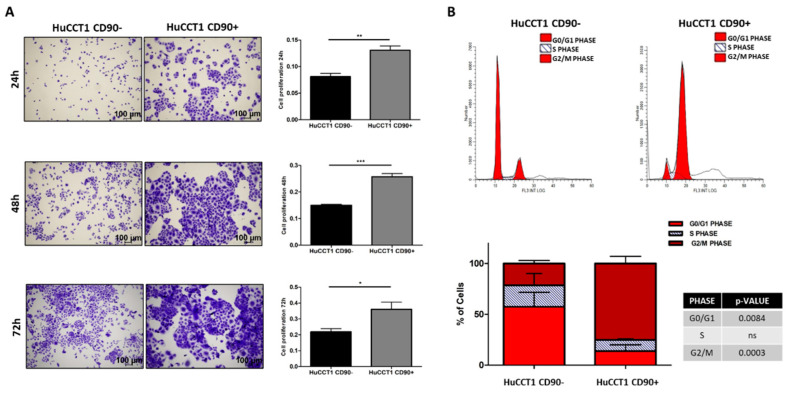
CD90 expression increased the cell turnover. (**A**) Proliferation assay performed on HuCCT1 CD90−/CD90+ cells for 24 h, 48 h, and 72 h in 2D culture. CD90 expression enhances cell proliferation in a time-dependent manner. Images were acquired with a microscope (Magnification: 4×; Scale bar, 100 µM), and the values reported in the graphs represent the mean ± SD of three independent experiments. (**B**) Cell cycle analysis by flow cytometry of HuCCT1 CD90−/CD90+ cells labelled with propidium iodide (PI). The percentage of cells in G2 phase was higher for HuCCT1/CD90+ cells, depicting a more proliferative profile than HuCCT1/CD90− cells. The calculated percentage of cell cycle distribution, as reported in the graph below, is presented as mean ± SD from three independent experiments. N.S., no significant difference. * *p* < 0.05; ** *p* < 0.01; *** *p* < 0.001, calculated with Student’s *t*-test.

To explain the predicted increase in hepatobiliary cancer by bioinformatic analysis, we investigated the proliferation and migration activity of HuCCT1, both CD90/+ and /CD90−. As reported in Figure 3A, HuCCT1/CD90+ cells were significantly more proliferative compared to HuCCT1/CD90− after 24, 48, and 72 h (*p* < 0.01, *p* < 0.001, and *p* < 0.05, respectively) in a 2D experimental model. Next, to further confirm the different proliferative efficiency between the two iCCA cell lines, we investigated cell cycle progression on HuCCT1/CD90+ and /CD90− cells by flow cytometry. As shown in Figure 3B, the percentage of HuCCT1/CD90+ cells in G2/M phase was 75.14%, while the percentage of HuCCT1/CD90− was 21.48%; this difference was statistically significant (*p* < 0.001). Consistently, the percentage of HuCCT1/CD90+ cells in G0/G1 phase was 13.79%, while that of HuCCT1/CD90− was 54.47%, again showing a statistically significant difference (*p* < 0.001).

**Figure 4:**
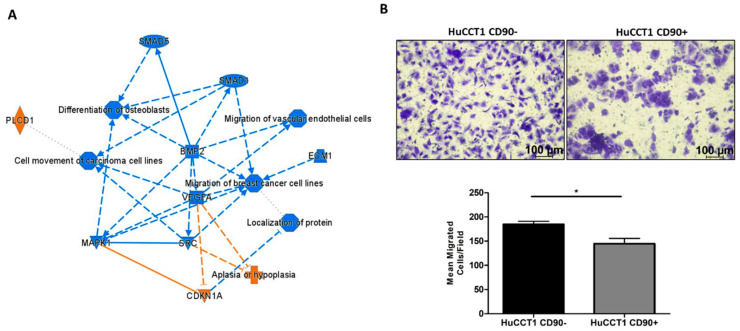
CD90 reduced cell migration. (**A**) Graphic summary of the iIngenuity pPathway aAnalysis (IPA), including canonical pathways, upstream regulators, and functions/diseases. (**B**). Blue denotes down-regulation and orange up-regulation. The migration of HuCCT1/CD90+ cells was significantly lower than of HuCCT1/CD90− cells. Representative images of migrated HuCCT1 CD90−/CD90+ cells were acquired with a microscope (Magnification: 10×, 100 µM), and the values reported in the graphs represent the mean ± SD of three independent experiments. * *p* < 0.05, calculated with Student’s *t*-test.

Pathways and functional classification analyses of DEGs performed with IPA indicated that various motile cellular functions were reduced in HuCCT1/CD90+, as shown in Figure 4A. To validate the bioinformatic prediction, we challenged HuCCT1 cells to migrate on Collagen I. As reported in Figure 4B, HuCCT1/CD90+ cells migrated less efficiently (*p* < 0.05) than HuCCT1/CD90− cells. In conclusion, HuCCT1/CD90+ were more proliferative but less motile than HuCCT1/CD90−.

### 2.3. CD90 Expression Is Associated with a More Epithelial Phenotype

**Figure 5:**
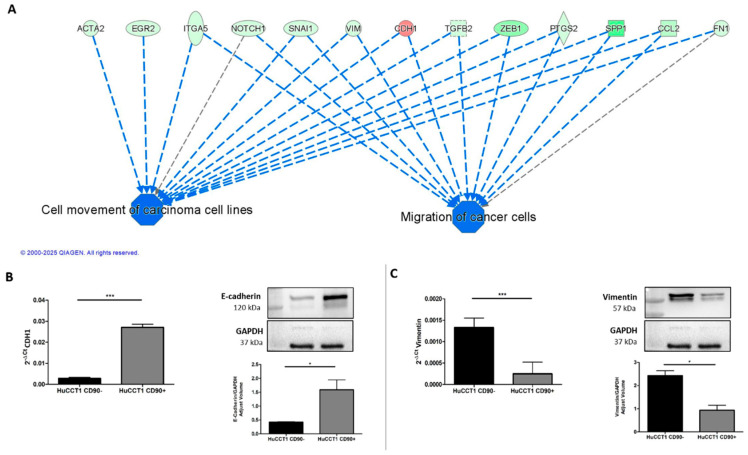
The expression of CD90 induced an epithelial cellular phenotype. (**A**) Migration of cancer cells was predicted as one of the most downregulated functions and its target molecules by IPA. Genes in red denote up-regulation and in green down-regulation. CD90 increased gene and protein expression of CDH1/E-Cadherin (**B**) and reduced Vimentin expression, both at the gene and protein levels (**C**). * *p* < 0.05; *** *p* < 0.001, calculated with Student’s *t*-test.

To further investigate the molecular mechanisms underlying the different migration capability of HuCCT1/CD90+ versus /CD90− cells, we first analyzed those genes mainly involved in cancer cell migration, as identified by IPA analysis. As reported in Figure 5A, CDH1 was the most upregulated gene; therefore, we investigated the expression of E-cadherin in both HuCCT1/CD90+ and /CD90− cells. As reported in Figure 5B, E-cadherin, both at the RNA and protein levels, was significantly (*p* < 0.001 and *p* < 0.05, respectively) more strongly expressed in HuCCT1/CD90+ compared to /CD90−. Conversely, Vimentin, both at the RNA and protein levels, was significantly (*p* < 0.001 and *p* < 0.05, respectively) more expressed in HuCCT1/CD90− than /CD90+ cells (Figure 5C). In conclusion, the higher expression of E-cadherin cell -cell junctions was more evident in the less migratory cells, while Vimentin was more expressed in the /CD90− cells.

**Figure 6:**
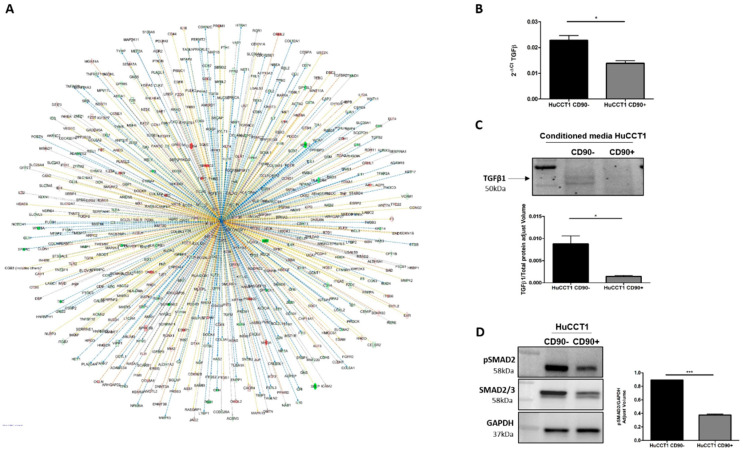
TGFβ-1 pathway activation was correlated to CD90. (**A**) TGF-β1 was predicted as an upstream regulator and its target molecules by IPA in HuCCT1/CD90+ vs. HuCCT1/CD90−. Genes in red were upregulated and in green downregulated. Lines in orange denote predicted activation; lines in blue denote predicted inhibition. TGF-β1 gene (**B**) and protein (**C**) expression was reduced in HuCCT1/CD90+ compared to HuCCT1/CD90−. (**D**) Activation of the TGF-β pathway, based on p-Smad2 protein expression, that was lower in cells with a high CD90 expression. * *p* < 0.05; *** *p* < 0.001, calculated with Student’s *t*-test.

A further bioinformatic pathway analysis predicted TGF-β1 as an upstream regulator of several deregulated genes (580) between HuCCT1/CD90+ vs. HuCCT1/CD90− cells, as shown in Figure 6A. To validate this finding, we investigated the TGF-β pathway in HuCCT1 CD90+/CD90− cells. As reported in Figure 6B, TGF-β1 gene expression was significantly (*p* < 0.05) downregulated in HuCCT1 CD90+ cells compared to HuCCT1/CD90− cells. Consistently, the levels of TGF-β1 protein were significantly (*p* < 0.05) less concentrated in the conditioned medium of HuCCT1/CD90+ cells than in /CD90− (Figure 6C). Finally, phospho(*p*)-Smad2 expression was also significantly (*p* < 0.05) reduced in HuCCT1/CD90+ compared to /CD90− cells (Figure 6D). In conclusion, HuCCT1/CD90+ cells express a lower level of TGF-β1 and consistently higher levels of E-cadherin, thus explaining the reduced migratory capability.

### 2.4. Galunisertib Overcomes CD90-Induced Resistance to Gemcitabine

**Figure 7:**
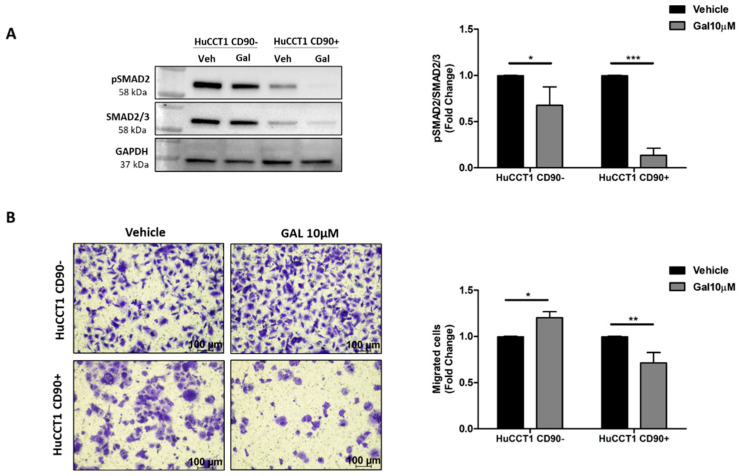
CD90 improved the efficacy of Galunisertib treatment. (**A**) Galunisertib reduced the TGF-β1/Smad pathway activation more strongly in HuCCT1/CD90+ cells compared to HuCCT1/CD90− cells, as observed by Western blot analysis. (**B**) Galunisertib inhibited cell migration only in HuCCT1/CD90+ cells. Migrated cells were counted in five random microscope fields for each transwell (Magnification: 10×; Scale bar, 100 µM). * *p* < 0.05; ** *p* < 0.01; *** *p* < 0.001, calculated with Student’s *t*-test.

To further confirm the role of TGF-β1 in modulating cell migration, we challenged HuCCT1/CD90+ with Galunisertib, a selective TGFβ-RI inhibitor, at a 10 µM concentration. As reported in Figure 7A, the drug treatment statistically (*p* < 0.001) dephosphorylated p-Smad2 in HuCCT1/CD90+ cells, while a lesser effect (although statistically significant) was observed on /CD90− cells. Next, Galunisertib strongly reduced HuCCT1/CD90+ cell migration (*p* < 0.01), while no effect was observed on HuCCT1/CD90− cells (Figure 7B).

**Figure 8:**
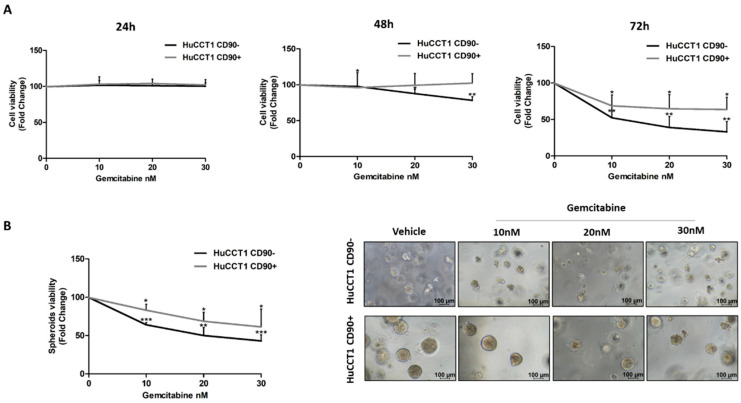
CD90 overexpression induced Gemcitabine resistance in iCCA cells. Viability assay was performed on HuCCT1 CD90−/CD90+ cells treated with increasing concentrations of Gemcitabine (10, 20, and 30 nM) for 72 h in 2D culture (**A**) and thirteen days in a 3D model (**B**). HuCCT1/CD90+ cells were chemoresistant compared to HuCCT1/CD90− cells that, instead, responded in a dose-dependent manner. Images of spheroids were acquired with a microscope (Magnification: 10×; Scale bar, 100 µM). * *p* < 0.05; ** *p* < 0.01; *** *p* < 0.001, calculated with Student’s *t*-test.

Finally, both HuCCT1/CD90+ and /CD90− cells were treated with Gemcitabine, a chemotherapeutic commonly used in patients with advanced iCCA. Gemcitabine used at concentrations ranging from 10 up to 30 nM more efficiently reduced (*p* < 0.01 and *p* < 0.01, respectively) cell viability of HuCCT1/CD90− compared to /CD90+ cells after 48 and 72 h (Figure 8A). These results were confirmed in the 3D-spheroid cultures (Figure 8B) previously characterized for CD90 expression (Figure S1).

**Figure 9:**
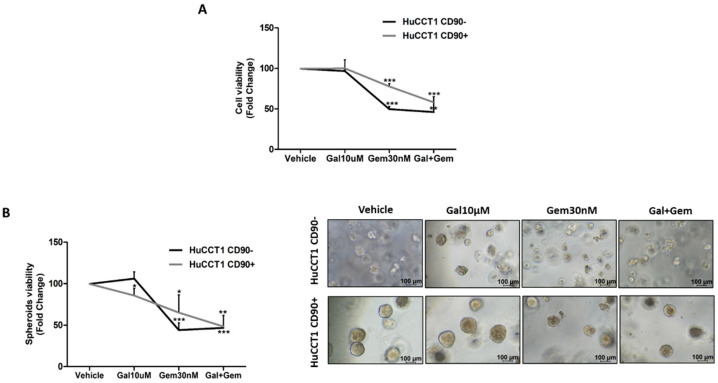
The Gemcitabine and Galunisertib combination had a synergistic effect in the presence of CD90. Both cell lines were treated with Gemcitabine (30 nM) and Galunisertib (10 µM) in monotherapy or combination. At the end of treatment, at 72 h and thirteen days in 2D (**A**) and 3D spheroids models (**B**), respectively, a proliferation assay was performed. The results show that the combined action of Gemcitabine and Galunisertib was only observed on HuCCT1/CD90+, whereas in HuCCT1/CD90− cells, there was no difference compared to monotherapy. Images of spheroids were acquired with a microscope (Magnification: 10×; Scale bar, 100 µM) * *p* < 0.05; ** *p* < 0.01; *** *p* < 0.001, calculated with Student’s *t*-test.

Finally, both HuCCT1/CD90+ and /CD90− cells were treated with Galunisertib, Gemcitabine, or both drugs in combination at the previously used concentrations. As reported in Figure 9A, Galunisertib used alone did not affect cell viability of either HuCCT1, while Gemcitabine was shown to more efficiently reduce (*p* < 0.001) cell viability of HuCCT1/CD90− compared to /CD90+ in the 2D experimental model previously described. However, the combination of both drugs had no synergic effect on HuCCT1/CD90−, whereas it significantly (*p* < 0.001) reduced cell viability of the HuCCT1/CD90+ cells. These results were also confirmed in 3D spheroid cultures (Figure 9B).

## 3. Discussion

Intrahepatic cholangiocarcinoma is a highly aggressive malignancy characterized by complex molecular alterations, including epigenetic dysregulation [25,26]. Among surface markers studied in cancer, CD90 is a cell surface glycoprotein that has been identified as a biomarker associated with an aggressive profile in multiple cancer types [27]. Recently, we reported that high CD90 expression in iCCA is linked to a worse prognosis [24]. However, its role in the progression of iCCA tumorigenesis is still not completely known. In this study, for the first time, we demonstrated how CD90 overexpression influences key biological aspects of iCCA cells, leading to enhanced proliferative properties and a reduced migratory potential. Specifically, we found that the overexpression of CD90 underpins distinct morphological changes and determines the following outcomes in iCCA HuCCT1 cell line: (1) an increased cell turnover; (2) a significant reduction in cell migration; (3) a downregulation of TGF-β1 signaling; and (4) resistance to Gemcitabine. Our results demonstrate that HuCCT1 cells with an elevated CD90 level exhibit shorter replication timing compared to cells expressing lower CD90, as previously described by Sukowati and colleagues in an HCC cell line [28], where CD90-positive cells displayed an increased proliferative rate. Furthermore, the overexpression of CD90 reduces HuCCT1 cell migration. To explain this phenotype, we performed in silico analysis of CD90 overexpressing cells, which revealed downregulation of TGF-β1. TGF-β1 is a well-known EMT inducer, driving tumor cells toward a mesenchymal phenotype, with enhanced migratory and invasive features, as observed in HCC cell lines [29,30]. Under EMT, cells lose their adhesive properties and cell–cell contact, undergoing cytoskeletal reorganization that ultimately leads to a fibroblast-like morphology [31]. In addition, bioinformatic analysis revealed increased E-cadherin and decreased vimentin levels in HuCCT1 CD90+ cells. This prediction was tested in our experimental plan. Specifically, the increased expression of E-cadherin and downregulation of vimentin highlight a more epithelial phenotype of the HuCCT1 overexpression. Consistently, HuCCT1/CD90+ cells also display a lower activation of the TGF-β1 pathway, a known master regulator molecule of the epithelial–mesenchymal transition, inversely correlated with E-cadherin expression [32]. Recently, the expression of CD90, a well-known stem cell biomarker, has been associated with resistance to different chemotherapeutic agents (cisplatin, cytarabine, etoposide, and paclitaxel) in various cancer cells, including those derived from lung, pancreatic, and gastric cancers [33]. Gemcitabine treatment for patients with unresectable iCCA offers limited benefits, especially due to the onset of drug resistance [34]. Intriguingly, Chen and colleagues reported that high levels of CD44, another stem cell marker associated with an invasive phenotype similar to CD90, are also linked to gemcitabine resistance in pancreatic cancer cells and preclinical models [35]. Based on this bulk of data, we hypothesized that CD90 could have a role in the resistance to Gemcitabine treatment in iCCA experimental models. Then, we tested our hypothesis by challenging HuCCT1 overexpressing CD90 in the presence of Gemcitabine and measured the drug effectiveness by means of cell viability using both 2D and 3D experimental models, which further supported the previous research findings. Our results strongly suggest that CD90 is associated with resistance to Gemcitabine treatment and that the TGF-β1 pathway is likely involved. Thus, we hypothesized that targeting the TGF-β1 pathway with Galunisertib could overcome the observed resistance of CD90+ cells to Gemcitabine. Galunisertib is no longer manufactured by Lilly company, as its development was discontinued due to internal policy, although it has a demonstrated efficacy compared to sorafenib as monotherapy, with an excellent safety profile [36]. Nevertheless, it remains a well-characterized and widely used preclinical tool for selective inhibition of TGF-β receptor I. In our study, Galunisertib was used to explore the CD90-dependent response to TGF-β1 blockade in iCCA, where it enhanced the synergistic effect of Gemcitabine. As shown in Figure 9A,B, cell viability assays under 2D and 3D conditions confirmed that Galunisertib monotherapy was non-cytotoxic at the tested concentration. We hypothesized that the effects observed with the combination treatment result from a specific CD90-mediated modulation of the TGF-β pathway.

In vitro experiments showed that Galunisertib itself had no significant effect on the viability of HuCCT1 cells, regardless of CD90 expression. However, when Gemcitabine was added, a synergistic effect was observed exclusively in HuCCT1/CD90+ cells.

In conclusion, our data demonstrate that CD90 promotes an aggressive phenotype in iCCA, characterized by an increased proliferative capacity and resistance to Gemcitabine. Nevertheless, the data obtained suggest that the CD90-TGF-β1 interaction could be an eligibility criteria to define responder and non-responder iCCA patients to the combined therapy. This may allow a further step towards personalized medicine care for patients with limited therapeutic options.

## 4. Materials and Methods

### 4.1. Cell Lines and Treatment

HuCCT1 CD90−/CD90+ human iCCA cell lines are cells transduced with human THY1-CMV-GFP and Lenti-CMV-GFP-2A-Puro-Blank Lentivirus (Aurogene, Rome, Italy) at MOI 40 [23]. Cells were monitored regularly with the MycoFluor™ Mycoplasma Detection Kit (ThermoFisher Scientific, Waltham, MA, USA) to obtain a mycoplasma-free cell population. The cell lines were cultured in RPMI supplemented with Sodium Pyruvate, Antibiotic–Antimycotic, Hepes, and 10% Fetal Bovine Serum (FBS) (Thermo Fisher Scientific, Waltham, MA, USA). Galunisertib (LY2157299, Selleckchem Chemicals, Houston, TX, USA) and Gemcitabine (Sigma-Aldrich, Gillingham, UK) were used in the in vitro studies.

### 4.2. Proliferation Assay

HuCCT1 CD90−/CD90+ cells were seeded in 24-well multiwell plates at a density of 8 × 10^3^ cells/well. After 24 h, 48 h, and 72 h cell seeding, cells were fixed in 4% PFA (pH 7.2 in PBS), stained with crystal violet for 10 min, and solubilized with 1% SDS for 4 h. One field per well was acquired in bright field at 4× magnification. Analysis was performed on the absorbance of cells acquired at 595 nm. Data are expressed as mean ± SD of three independent biological experiments.

### 4.3. Flow Cytometric Analysis of Cell Cycle

For cell cycle analysis, HuCCT1 CD90−/CD90+ cells were cultured on 6-well multiwell plates (Corning, Bedford, MA, USA) for 3 days. Cell cycling was performed on cell suspensions obtained after trypsinization. After cell counting, 1 × 10^6^ cells were considered. The cells were washed once in cold PBS, fixed with cold 70% ethanol, and kept at 4 °C overnight. The next day, the fixed cells were washed twice in PBS. After discarding the supernatant, the cells were treated with RNase A (Sigma, St. Louis, MO, USA; 100 µg/mL) for 15 min at 37 °C to remove RNA contamination. Next, propidium iodide (Sigma, St. Louis, MO, USA) was added to the cells (200 µL from a 50 µg/mL stock solution), and they were incubated for 30 min at 4 °C in the dark. Finally, the DNA content of the cells was analyzed by flow cytometry to determine the distribution of cells at different stages of the cell cycle.

### 4.4. Transwell Migration Assay

For the migration assay, cell culture inserts with 8 µm pores (Corning, Inc., Bedford, MA, USA), suitable for 24-well plates, were used. The down side of the membrane of the inserts was coated with a solution of rat tail collagen I at a concentration of 10 µg/mL for 2 h at room temperature (Gibco, Carlsbad, CA, USA). Then, 25 × 10^3^ cells, treated or not with Galunisertib, were resuspended in serum-free RPMI and seeded in the upper chamber of the transwell, while complete medium was added to the lower chamber. The cells were allowed to migrate for 17 h at 37 °C and 5% CO_2_. After incubation, the cells were fixed in 4% PFA (pH 7.2 in PBS) for 10 min and stained with crystal violet for 10 min. Excess dye was removed by washes with distilled water. Five fields per membrane were acquired at 10× magnification. Cell migration capacity was determined by counting the number of migrated cells/field. Data are expressed as mean ± SD of three independent experiments.

### 4.5. Spheroid Formation Assay

Matrigel Matrix (Corning, Bedford, MA, USA) was coated on 96-well plates. Refrigerated Matrigel was released into the multiwells that were kept on ice and placed in an incubator at 37 °C to allow gelation. Subsequently, 3 × 10^3^ cells/well were resuspended in refrigerated Matrigel to allow embedding within the matrix. The plates were then incubated at 37 °C to ensure Matrigel gelation. The culture was maintained for 5 days to allow the spheroids to grow. Subsequently, the spheroids were treated or not with Gemcitabine (10, 20, and 30 nM) or Galunisertib (10 µM) in monotherapy or in combination for 7 days. At the end of the treatment period, a proliferation assay was performed, namely CellTiter 96^®®^ AQueous One Solution Cell Proliferation Assay (Promega Italia s.r.l., Milan, Italy), a colorimetric method to determine cell viability.

### 4.6. Spheroid Immunofluorescence

As a first step, the spheroids included in the Matrigel were fixed in a 4% PFA solution at 4 °C for 30 min, after which two washes were performed with 0.1% Triton X-100 in TBS. The samples were permeabilized with 0.5% Triton X-100 in TBS at room temperature for 1 h. Subsequently, the spheroids were incubated with the primary anti-THY1 antibody (1:150, Cell Signaling Technology, Danvers, MA, USA) in a buffer solution (2% bovine serum albumin/Triton X-100 0.1% in TBS) overnight at 4 °C in rotation. The next day, after the washes, the samples were incubated with the secondary anti-mouse G H&L antibody (1:50 Alexa Fluor 488, Thermo Fisher Scientific, Waltham, MA, USA) for 3 h at room temperature in rotation and in the dark. After further washing, to stain the nuclei, the spheroids were labelled with 0.5 ng/mL PureBlu DAPI nuclear dye (Bio-Rad Laboratories, Hercules, CA, USA) in 0.1% Triton X-100 in TBS for 1 h at room temperature and in the dark. After incubation, the spheroids were placed on slides and coated with ProLong™ Diamond Anti-Fading Mounting Medium (Invitrogen from ThermoFisher Scientific, Waltham, MA, USA). Confocal images of the spheroids were acquired using a Nikon Ti2-E (Nikon Co., Tokyo, Japan) inverted research microscope equipped for confocal imaging in combination with a Nikon A1rSi Laser Point Scanning confocal system, Plan Fluor Ph 20× objective, and NIS-Elements AR 5.0 software and enhanced with a deconvolution method based on the Richardson–Lucy algorithm.

### 4.7. Immunofluorescence

Cells were seeded in chamber slides to analyze the expression of F-actin by immunofluorescence staining. Cells were fixed with PFA 4% and permeabilized with 0.1% Triton X-100 in PBS in 2% Bovine Serum Albumin for 30 min. Subsequently, cells were incubated with primary antibody Rhodamine Phalloidin TRITC (1:100, Sigma, St. Louis, MO, USA) for 1 h. After three washes in PBS, cells were incubated with secondary immunoglobulin G H&L (Alexa Fluor 488, Thermo Fisher Scientific, Waltham, MA, USA) for 1 h. Finally, the nuclei were stained with 4′,6-diamidino-2-phenylindole (DAPI)-supplemented antifade mounting medium VECTASHIELD (Vector Lab, Burlingame, CA, USA). Images were acquired using The Eclipse Ti2 microscope (Nikon Inc., Melville, NY, USA) at 20× magnification.

### 4.8. Viability Assay

Cells were seeded in 96-well plates at a concentration of 3 × 10^3^ cells/well and maintained at 37 °C 5%CO_2_. The next day, cells were treated or not with Gemcitabine (10–20–30 nM) at 24 h, 48 h, and 72 h or Galunisertib (10 µM) in monotherapy or in combination for 72 h. At the end of the experiment, a proliferation assay was performed using CellTiter 96^®^ AQueous One Solution Cell Proliferation Assay (Promega Italia s.r.l., Milan, Italy) to evaluate cell viability.

### 4.9. RNA Extraction

Total RNA was extracted with an miRNeasy mini kit (Qiagen, Hilden, Germany) according to the manufacturer’s instructions. RNA concentration was determined with the Qubit™ RNA HS Assay kit (Thermo Fisher Scientific, Waltham, MA, USA) on a Qubit Fluorometer (Thermo Fisher Scientific, Waltham, MA, USA). RNA quality was evaluated using High Sensitivity RNA ScreenTape (Agilent Technologies, Palo Alto, CA, USA) on an Agilent 4200 TapeStation system (Agilent Technologies, Palo Alto, CA, USA).

### 4.10. Whole Transcriptome Profiling

Total RNA samples were reverse-transcribed using the Ion Torrent™ NGS Reverse Transcription Kit (Thermo Fisher Scientific, Waltham, MA, USA) according to the manufacturer’s instructions. Amplification was performed using the Ion AmpliSeq Transcriptome Human Gene Expression core panel (Thermo Fisher Scientific, Waltham, MA, USA) on the Ion Chef System. The barcoded libraries were quantified by qPCR with the Ion Library TaqMan Quantitation kit (Thermo Fisher Scientific, Waltham, MA, USA). Then, libraries were templated on the Ion Chef and sequenced using a 540 chip on the Ion GeneStudio S5 Prime system (Thermo Fisher Scientific, Waltham, MA, USA).

Sequencing data are available under accession number GSE294148 at the Gene Expression Omnibus https://www.ncbi.nlm.nih.gov/geo/query/acc.cgi?acc=GSE294148) (accessed on 8 April 2025).

### 4.11. Quantitative Real-Time Reverse Transcription (qRT-PCR)

cDNA was obtained from 2 μg of total RNA using the High-Capacity cDNA Reverse Transcription Kit (Applied Biosystems by Thermo Fisher Scientific, Waltham, MA, USA) according to the manufacturer’s instructions. Quantitative PCR reactions were performed using iTaq Universal SYBR Green Supermix (Biorad Laboratories, Hercules, CA, USA) and primers for TGFB (qHsaCID0017026) (Biorad Laboratories, Hercules, CA, USA), CDH1 (qHsaCID0015365) (Biorad Laboratories, Hercules, CA, USA), VIM (qHsaCED0042034) (Biorad Laboratories, Hercules, CA, USA), and GAPDH (qHsaCED0038674) (Biorad Laboratories, Hercules, CA, USA). Experiments were repeated three times in triplicate. Relative expression was calculated using the 2^−ΔΔCt^ method.

### 4.12. Western Blotting

Protein expression was evaluated on purified cell lysates and concentrated conditioned media. Total cellular proteins were extracted using T-PER tissue protein extraction reagent (ThermoFisher Scientific, Waltham, MA, USA) with Halt protease and phosphatase inhibitor (ThermoFisher Scientific, Waltham, MA, USA). Proteins were separated in 4–20% Tris-glycine sodium dodecyl sulfate polyacrylamide gels (Bio-Rad Laboratories, Hercules, CA, USA). Membranes were incubated with the following primary antibodies: anti-TGFβ1 (1:1000, Invitrogen, Waltham, MA, USA); anti-phosphoSmad2 (1:1000, Abcam, Cambridge, UK), anti-Smad2/3 (1:1000, Cell Signaling Technology, Danvers, MA, USA), anti-E-cadherin (1:1000, Cell Signaling Technology, Danvers, MA, USA), anti-Vimentin (1:1000, Cell Signaling Technology, Danvers, MA, USA), and anti-glyceraldehyde-3-phosphate dehydrogenase (GAPDH) (1:1000, Santa Cruz Biotechnology, Santa Cruz, CA, USA). After overnight incubation with the primary antibody, membranes were washed and then incubated with secondary antibodies: anti-rabbit (1:2000, Cell Signaling Technology) or anti-mouse (1:2000, Biorad Laboratories, Hercules, CA, USA). The chemiluminescence signal of proteins was revealed using Clarity Max Western ECL Substrate (Bio-Rad) and captured with the ChemiDoc MP instrument (Bio-Rad Laboratories, Hercules, CA, USA) using Image Lab 5.2.1. The relative density of the bands was calculated using ImageLab software.

### 4.13. Bioinformatics and Statistical Analyses

Ion Torrent Suite Server v5.16.1 (Thermo Fisher Scientific, Waltham, MA, USA) software was used to generate the transcription data as raw read counts using the ampliSeqRNA plugin. Differential expression analyses were performed in the Transcriptome Analysis Console 4.0 software (Thermo Fisher Scientific, Waltham, MA, USA). Differentially expressed genes (DEGs) were determined using the Limma eBayes method with a threshold of fold-change of 1.5 and *p*-value ≤ 0.05. Hierarchical clustering was generated with Alt Analyze 2.1.3 software [37]).

Canonical pathways, biological processes, and molecular networks associated with differentially expressed genes were analyzed using Ingenuity Pathway Analyses (IPA) software Version 24.0.2 (Qiagen, Germantown, MD, USA). All biological replicates, presented as mean ± standard error, were analyzed with Student’s *t*-test using statistical software Graph Pad Prism version 5.0 (La Jolla, CA, USA).

## 5. Conclusions

In conclusion, we emphasize that CD90 confers aggressiveness in iCCA and that the interaction with TGF-β1 could be a characteristic on which to stratify patients with iCCA who are suitable for combination therapy, paving the way toward the adoption of new personalized therapeutic strategies. Notably, Galunisertib monotherapy showed no significant effect on cell viability, whereas its combination with Gemcitabine led to a synergistic response, specifically in /CD90+ cells, underscoring the value of combinatorial approaches in overcoming drug resistance in aggressive iCCA.

## Data Availability

The original contributions presented in this study are included in the article/Supplementary Materials. Further inquiries can be directed to the corresponding author. Sequencing data are available under accession number GSE294148 at the Gene Expression Omnibus (https://www.ncbi.nlm.nih.gov/geo/query/acc.cgi?acc=GSE294148) (accessed on 8 April 2025).

## Supplementary Materials

The following supporting information can be downloaded at https://www.mdpi.com/article/10.3390/ijms26114973/s1.

## Abbreviations

iCCA	Intrahepatic cholangiocarcinoma
CD90	Cluster Differentiation 90
TGF-β1	Transforming Growth Factor-beta 1
DEG	Differentially expressed genes
IPA	Ingenuity Pathway Analyses
EMT	Epithelial–mesenchymal transition
