# Supplementary figures and images for: Inhibiting the TGF-β1 Pathway Reduces the Aggressiveness of Intrahepatic CCA HuCCT1 CD90-Positive Cells
Source: Int J Mol Sci. 2025 May 22;26(11):4973. doi: 10.3390/ijms26114973 (PMC12155448; doi:10.3390/ijms26114973)

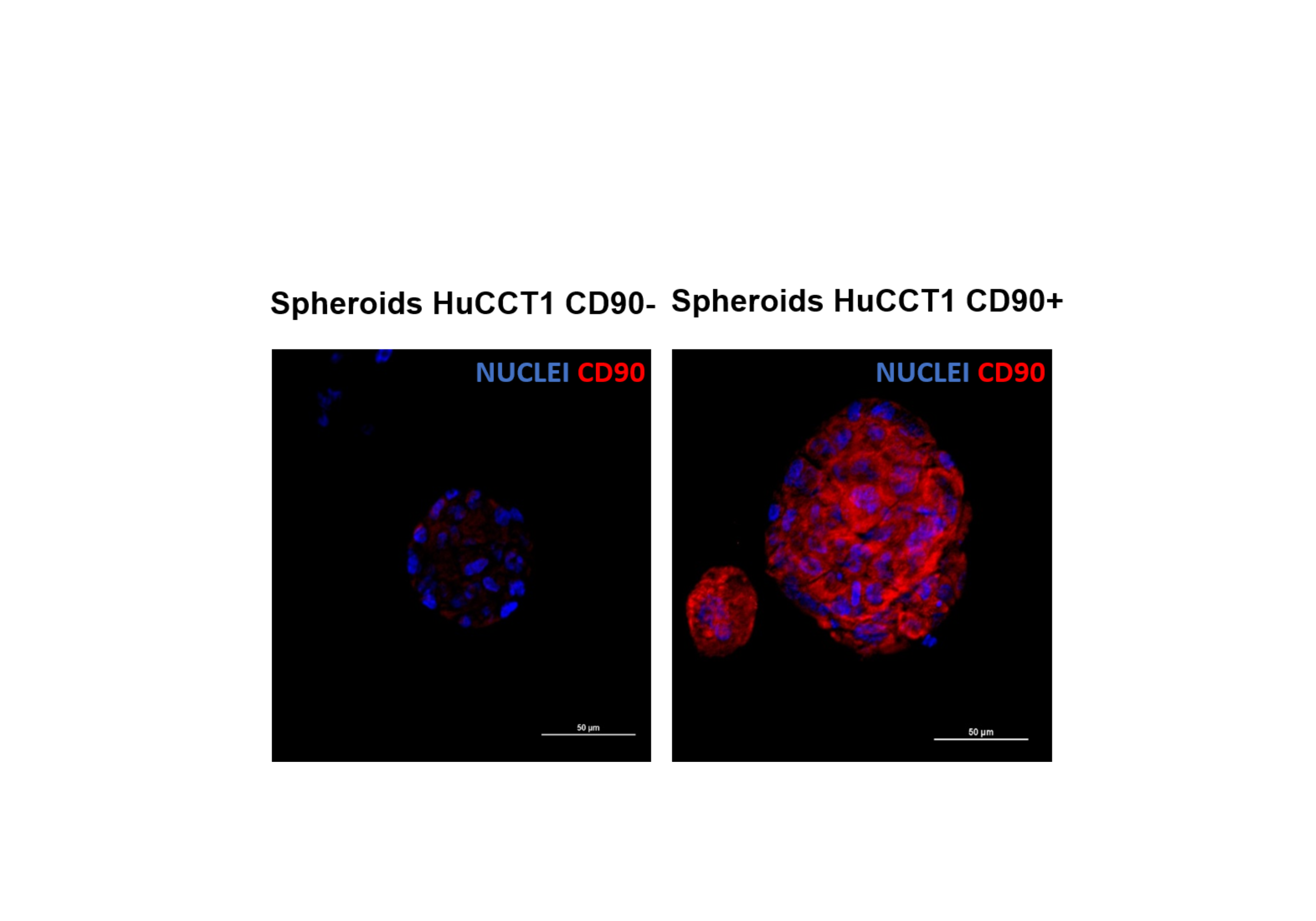

Supplement: Supplementary file 1 [file ijms-26-04973-s001.zip › ijms-3606927-supplementary.png]
